# Antimicrobial Resistance and Virulence of Methicillin-Resistant *Staphylococcus* *aureus* from Human, Chicken and Environmental Samples within Live Bird Markets in Three Nigerian Cities

**DOI:** 10.3390/antibiotics9090588

**Published:** 2020-09-08

**Authors:** Flora Olubunmi Ogundipe, Olufemi Ernest Ojo, Andrea T. Feßler, Dennis Hanke, Olajoju Jokotola Awoyomi, David Ajiboye Ojo, Aderonke Kofoworola Akintokun, Stefan Schwarz, Sven Maurischat

**Affiliations:** 1 Department of Microbiology, College of Biosciences, Federal University of Agriculture, Abeokuta 110124, Nigeria; flora.ogundipe@yabatech.edu.ng (F.O.O.); daojo3@yahoo.com (D.A.O.); ron_akintokun@yahoo.com (A.K.A.); 2 Institute of Microbiology and Epizootics, Centre for Infection Medicine, Department of Veterinary Medicine, Freie Universität Berlin, 14163 Berlin, Germany; andrea.fessler@fu-berlin.de (A.T.F.); dennis.hanke@fu-berlin.de (D.H.); 3 Department of Veterinary Microbiology, College of Veterinary Medicine, Federal University of Agriculture, Abeokuta 110124, Nigeria; ojooe@funaab.edu.ng; 4 Department of Veterinary Public Health and Preventive Medicine, College of Veterinary Medicine, Federal University of Agriculture, Abeokuta 110124, Nigeria; jojuawoyomi@yahoo.com; 5 Department Biological Safety, German Federal Institute for Risk Assessment (BfR), 10589 Berlin, Germany; sven.maurischat@bfr.bund.de

**Keywords:** CA-MRSA, multidrug resistance, Nigeria, resistance genes, sequence types, SCC*mec*, *spa* types, *dru* types, virulence genes

## Abstract

**Background:** Methicillin-resistant *Staphylococcus aureus* (MRSA) has emerged as a major threat to public health. This study investigated the occurrence of MRSA in humans, chickens, chicken meat and environmental samples within poultry farms and live bird markets in southwestern Nigeria. **Methods:** MRSA were isolated using selective culture and tested for antimicrobial susceptibility by broth microdilution. Selected isolates were characterized by whole genome sequencing (WGS). From WGS data, *spa*, *dru*, multilocus sequence typing (MLST) and SCC*mec* types, but also virulence and antimicrobial resistance genes, were identified. **Results:** Fifty-six MRSA isolates were detected in 734 samples. They showed resistance to β-lactams (100%), tetracycline (60.7%), ciprofloxacin (33.9%), erythromycin (28.6%), gentamicin (32.1%), and trimethoprim/sulfamethoxazole (10.7%). All 30 isolates investigated by WGS carried *mecA*, *dfrG*, and *tet*(38) genes. Other resistance genes detected were *blaZ* (83.3%), *fosB* (73.3%), *tet*(K) (60.0%), *aacA-aphD* (36.6%), *aphA3* (33.3%), *msr*(A) (30.0%), *mph*(C) (30.0%), *dfrS1* (3.3%), and *sat4* (3.3%). Seven *spa* types (t091, t314, t657, t1476, t2331, t4690 and t12236), four known (dt9aw, dt10ao, dt10cj, and dt11a) and two novel (dt10dr and dt11dw) *dru* types, as well as five sequence types (ST8, ST121, ST152, ST772 and ST789) were found among the MRSA isolates. All ST121 isolates carried an SCC*mec* type IV cassette and were not *dru*-typeable. ST152 and ST121 were found only in specific sample categories within defined locations, while ST8 and ST772 were distributed across most sample categories and locations. Three SCC*mec* types, IVa, V and Vc, were identified. All MRSA isolates possessed virulence genes including *aur*, *clpP*, *coa*, *fnbA*, *esaA*, *hly*, *hla*, *ica*, *isdA*, *srtB*, *sspA*, and *vWbp*, among others. The toxic shock syndrome toxin gene (*tst*) was not detected in any isolate, whereas the Pantone–Valentine leukocidin genes *lukF-PV*/*lukS-PV* were present in all ST121, all ST772, and all but one ST152 isolates. **Conclusion:** The results of this study (i) showed that chicken meat is contaminated by MRSA and (ii) suggested that live bird markets may serve as focal points for the dissemination of MRSA within the community.

## 1. Introduction

*Staphylococcus aureus* is an opportunistic pathogen that can cause significant health challenges in humans and animals [1]. Because of the ubiquity of *S*. *aureus*, it is difficult to prevent the involvement of the organism as secondary tissue invader following trauma or primary infections [2]. The presence of virulence factors associated with bacterial adhesion, tissue invasion, immune evasion and toxin production may account for the frequent involvement of *S. aureus* in clinical infections and food poisoning [3]. Over the years, *S. aureus* has acquired a wide variety of antimicrobial resistance properties, including methicillin resistance. The emergence and spread of methicillin-resistant *S. aureus* (MRSA) strains that showed not only broad spectrum resistance to β-lactam antimicrobial agents, but often also resistance to multiple other antimicrobial agents, constitutes a great threat to the usefulness of antimicrobial agents in the treatment of staphylococcal infections, although it could lead to an increased risk of infection spread, higher medical cost and sometimes mortality [4]. It is therefore not surprising that MRSA is one of the leading public health concerns worldwide.

MRSA first emerged as a nosocomial pathogen among hospitalized patients in healthcare facilities, but was later reported in community infections as well as in asymptomatic carriers among human populations. The subsequent detection of livestock-associated MRSA (LA-MRSA) in farm animals generated an increased interest with a OneHealth perspective [5]. It also became pertinent to understand factors that play critical roles in the increasing incidence of the organism in different human, animal, food and environmental sources from different geographical locations. Regrettably, there are few data about MRSA, its molecular properties and the transmission routes, especially via meat production and marketing chains from low-income countries, like Nigeria. Earlier studies under separate investigations have documented the detection of MRSA in samples of human [6,7] and animal origin [8,9]. Continuous surveillance using the latest technology will provide in-depth analysis of MRSA profiles for a better understanding of the origins and dissemination patterns in human and animal populations across different geographical locations. The present study, therefore, investigated the presence and potential interrelatedness of MRSA in human, chicken and environmental samples from live bird markets in three cities located in southwestern Nigeria.

## 2. Results

### 2.1. Identification of Staphylococcus aureus

Sixty-one isolates were confirmed as *S. aureus* among 636 putative staphylococcal isolates screened by MALDI-TOF MS and/or RT-PCR. Thirty-one of the confirmed isolates originated from chicken meat (freshly dressed chicken meat and imported/frozen chicken meat), 22 isolates were from humans (chicken meat processors and consumers) and eight originated from environmental samples (table and knife swabs) (Table 1). *S. aureus* was not detected in samples from on-farm chickens. Fifty-six of 61 *S. aureus* were categorized as MRSA based on phenotypic resistance to oxacillin and the presence of the *mecA* gene. The MRSA isolates included those from chicken meat (30/56; 53.6%), humans (18/56; 32.1%) and environmental samples (8/56; 14.3%).

### 2.2. Antimicrobial Susceptibility

According to the Clinical Laboratory Standards Institute (CLSI) [10], MRSA isolates are considered as resistant to all β-lactam antibiotics—except specific anti-MRSA β-lactams, regardless of their minimal inhibitory concentrations (MIC) values. The MIC values of the 16 non-β-lactam antimicrobial agents tested are displayed in Table 2 for all 56 MRSA isolates. All 56 MRSA isolates were susceptible to clindamycin, quinupristin/dalfopristin, linezolid, and vancomycin, but showed varying degrees of resistance to other antimicrobial agents (Table 2), including resistances to tetracycline (60.7%), ciprofloxacin (33.9%), enrofloxacin (33.9%), gentamicin (32.1%), erythromycin (28.6%), and trimethoprim/sulfamethoxazole (10.7%). Isolates from chicken meat showed higher percentages of resistance to tetracycline (83.3%) and trimethoprim/sulfamethoxazole (16.6%) than isolates from humans (5.6% for each of tetracycline and sulfamethoxazole/trimethoprim). In contrast, isolates from humans had higher percentages of resistance to ciprofloxacin (94.4%), gentamicin (88.9%) and erythromycin (83.3%) than isolates from dressed chicken (ciprofloxacin 6.7%, gentamicin 6.7% and erythromycin 3.3%). Among the isolates from chicken meat, only the two isolates from imported/frozen chicken meat were resistant to ciprofloxacin and gentamicin. In total, 25 out of the 56 MRSA isolates displayed a multidrug resistance phenotype, i.e. resistance to three or more classes of antimicrobial agents [11]. Multidrug-resistant isolates were predominantly from human samples (17/25; 68.0%). Six isolates from freshly dressed chicken meat (6/25; 24.0%) and two (2/25; 8.0%) from imported/frozen chicken meat were also multidrug-resistant. The observed multidrug resistance profiles were BLA-CIP-SXT-TET (one isolate) and BLA-CIP-ERY-GEN (16 isolates) detected among human isolates, BLA-SXT-TET (five isolates) and BLA-ERY-TET (one isolate) from freshly dressed chicken meat, as well as BLA-CIP-GEN-TET (two isolates) from imported/frozen chicken meat.

### 2.3. Multilocus Sequence Typing and Core Genome Multilocus Sequence Typing

**Figure 1:**
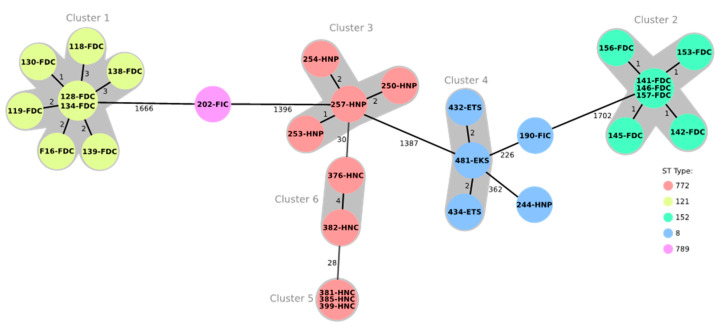
The minimum spanning tree showing the clonal relationship of 30 MRSA isolates based on a core genome multilocus sequence typing (cgMLST) analysis including 1783 genes and using the Ridom SeqSphere+ software. Each circle represents an allelic profile and the connecting lines display the number of different alleles between the distinct profiles. The sequence types (ST) are indicated by color, while the individual isolate IDs are shown within the circles. FDC: Freshly dressed chicken; FIC: Frozen/imported chicken; HNP: Human nasal swab, processor; HNC: Human nasal swab, consumer; ETS: Environment, table swab; EKS: Environment, knife swab.

Multilocus sequence typing (MLST) results, as deduced from the whole genome sequences, showed five sequence types (STs) among the 30 tested isolates (Table 3). The detected sequence types were ST772 (9/30; 30.0%), ST121 (8/30; 26.7%), ST152 (7/30; 23.3%), ST8 (5/30; 16.7%), and ST789 (1/30; 3.3%). ST8 isolates were obtained from human (one isolate from a meat processor, Abeokuta), environmental (two isolates from table swabs, Ibadan and one isolate from a knife swab, Abeokuta) and chicken meat samples (one isolate from frozen/imported chicken, Abeokuta). All other human isolates, irrespective of the geographical origin, belonged to ST772. All isolates from freshly dressed chicken meat in Abeokuta belonged to ST121, while the corresponding isolates from Ibadan belonged to ST152. The only ST789 isolate was from frozen/imported chicken meat in Abeokuta. Core genome multilocus sequence typing (cgMLST) analysis including 1783 genes showed six clusters among the 30 whole genome sequenced isolates (Figure 1). These clusters were strongly associated with the different STs. Cluster 1 comprised the ST121 isolates from freshly dressed chickens in Abeokuta, while cluster two contained the corresponding ST152 isolates from Ibadan. Clusters 3, 4 and 5 included the ST772 isolates from nasal swabs of processors and consumers in Abeokuta and Lagos. Cluster 4 comprised the ST8 isolates obtained from swabs of knives and tables. Finally, there were two ST8 isolates, one from the nasal swab of a processor and the other from a frozen/imported chicken, which differed from the center isolate of cluster 4 by 226 and 362 alleles, respectively. The ST789 isolate was distantly related to the isolates of cluster 1 and cluster 3, with 1666 and 1396 differing alleles, respectively. Within each cluster, the number of varying alleles ranged between 0 and 4 (Figure 1).

### 2.4. Staphylococcal Cassette Chromosome mec (SCCmec) Typing

Three types of SCC*mec* elements (SCC*mec* IVa, V and Vc) were recognized among the 30 MRSA isolates subjected to whole genome sequencing (WGS). All ST121 isolates had an SCC*mec* IVa element, while all ST772 isolates and the single ST789 isolate harbored SCC*mec* V. SCC*mec* Vc was found in isolates belonging to ST8 and ST152 (Table 3).

### 2.5. Staphylococcus aureus Protein A (spa) and Direct Repeat Unit (dru) Typing

Seven different unrelated *spa* types were detected among the 30 MRSA isolates analyzed by WGS (Table 3). The *spa* types were t657 (*n* = 9), t314 (*n* = 8), t4690 (*n* = 7), t12236 (*n* = 3), t091, t1476 and t2331 (*n* = 1, each). Isolates from chicken meat had four *spa* types: t314 (all eight isolates from freshly dressed chicken meat, Abeokuta), t4690 (all seven isolates from freshly dressed chicken meat, Ibadan), t1476 and t091 (one isolate each from frozen/imported chicken meat, Abeokuta). There were two *spa* types detected among human isolates, as follows: t2331 (one isolate from meat processor, Abeokuta), t657 (four isolates from meat processors, Abeokuta and five isolates from meat consumers, Lagos). All three tested isolates from table swabs or knife swabs had *spa* type t12236. ST8 was associated with three *spa* types (t1476, t2331, and t12236), whereas other STs had distinct *spa* types (Table 3).

Four known and two novel *dru* types were identified among 22 of the 30 sequenced isolates. All eight ST121/t314/SCC*mec* IVa isolates were not *dru*-typeable. In these isolates, the region between the 3′ end of the *mecA* gene and the following IS*431* element, which commonly contains the *dru* region, comprised only a relic of 218 bp. This relic did not include parts of the variable *dru* region, which is used for *dru* typing. The four already known *dru* types included dt9aw (*n* = 2), dt11a (*n* = 9), dt10ao (*n* = 7), and dt10cj (*n* = 2). The *dru* type dt9a was present in single ST152 and ST8 isolates, while the two closely related *dru* types dt10ao and dt10cj were exclusively present in ST772 isolates. MRSA displaying dt11a included six ST152, two ST8 and the single ST789 isolate. The two new *dru* types, assigned by the *dru* typing database as dt10dr (repeat order: 5a-2d-4a-0-2d-5b-3a-3o-3b-4e] and dt11dw [repeat order: 5a-2d-4a-0-2d-5b-2a-2g-2c-4e-3e) were found in ST8 isolates of different origins (Table 3).

### 2.6. Virulence Genes

All tested isolates had virulence genes associated with adhesion and biofilm production (*fnbA*, *icaA*, *icaB*, *icaC*, *icaD* and *icaR*), cell lysis and tissue invasion (*aur*, *clpP*, *coa*, *esaA*, *esaB*, *geh*, *lip*, *sspA*, *sspB*, *sspC*, *vWbp*), blood cell lysis (*hla*, *hlb*, *hld*, *hlgB*, *hlgC*, *hly*), immune evasion (*cap*, *chp*, *spa*, *sbi*, *scn*) and iron uptake (*strB*, *isdA*, *isdB*, *isdC*, *isdD*, *isdE*, *isdF*, *isdG*) [14,15]. Other virulence genes, such as *clfA*, *clfB*, *cna*, *ebp*, *esxB*, *fnbB*, *hysA*, *lukF-PV*, *lukS-PV*, *lukD*, *lukE*, *map*, *sak*, *sdrC*, *sdrD*, *sea*, *seb*, *sec* and *sell*, were detected among the isolates at varying frequencies (Table 4). Genes encoding toxic shock syndrome toxin-1 (*tst*) as well as staphylococcal enterotoxin-like toxins K (*selk*) and Q (*selq*) were absent in all tested isolates.

### 2.7. Antimicrobial Resistance Genes and Resistance-Mediating Mutations

Twelve antimicrobial resistance genes were identified in the 30 isolates examined by WGS. All these isolates carried the methicillin resistance gene *mecA*, the trimethoprim resistance gene *dfrG*, and the tetracycline resistance gene *tet*(38). Other resistance genes present in the isolates were *blaZ* (83.3%), *fosB* (73.3%), *tet*(K) (60.0%), *aacA-aphD* (36.6%), *aphA3* (33.3%), *msr*(A) (30.0%), *mph*(C) (30.0%), *dfrS1* (3.3%), and *sat4* (3.3%). In addition to the aforementioned complete resistance genes, truncated *sat4* and truncated *aadE* genes were found in nine and ten MRSA isolates, respectively. Eight different resistance gene combinations were detected among the isolates (Table 5).

Despite the observation that the tetracycline resistance gene *tet*(38) was present in all 30 isolates, only 18 isolates showed phenotypic tetracycline resistance with tetracycline MICs ranging between 32 and 256 mg/L. Since all 18 phenotypically resistant isolates co-harbored the tetracycline resistance gene *tet*(K), it is likely that *tet*(K) is the gene responsible for the tetracycline resistance phenotype observed. Among the ten erythromycin-resistant isolates, nine carried the genes *msr*(A)-*mph*(C), whereas the remaining isolate 145 neither carried a known acquired macrolide resistance gene, nor mutations in 23S rRNA. All 11 gentamicin-resistant isolates carried the gene *aacA*/*aphD* and varied in their MICs between 32 and ≥512 mg/L. Ten of the whole genome sequenced MRSA carried the aminoglycoside phosphotransferase gene *aphA3*, which confers resistance to kanamycin and neomycin. Although only neomycin was tested in this study and there are no approved neomycin clinical breakpoints, all ten MRSA exhibited neomycin MICs of ≥128 mg/L, which suggested that this gene is active. The remaining 20 MRSA had distinctly lower neomycin MICs of 0.25–0.5 mg/L.

All 12 fluoroquinolone-resistant isolates with ciprofloxacin, enrofloxacin and nalidixic acid MICs of 8–≥32 mg/L, 4–≥32 mg/L and 128–≥256 mg/L had one mutation each in the quinolone-resistance determining regions of the genes *gyrA* and *grlA*. These mutations resulted in the deduced amino acid sequence of GyrA to the substitution Ser84Leu and in the corresponding sequence of GrlA, either to the substitution Ser80Tyr (isolates 190, 250, 253, 254, 257, 376, 381, 382, 385, and 399), or to the substitution Ser80Phe (isolates 202 and 244).

Analysis of the five sulfonamide-trimethoprim-resistant isolates 119, 141, 142, 146 and 244 for mutations in the *folP* gene that have been described to result in sulfonamide resistance [16], revealed that only isolate 244 had two mutations that led to the FolP amino acid substitutions Phe17Leu and Glu208Lys. Although all isolates carried the trimethoprim resistance gene *dfrG*, neither one nor two trimethoprim resistance genes alone can confer resistance to the sulfonamide/trimethoprim combination. In this study, the isolate 244 had an SXT MIC of 16/304 mg/L and harbored both *folP* mutations associated with sulfonamide resistance and the *dfrG* gene.

## 3. Discussion

This study showed the presence of MRSA in human, chicken meat and environmental samples from live bird markets/chicken slaughter centers in the study locations. No *S. aureus* was detected in samples from live chickens on farms, which could be due to the limited number of farms investigated. Only three farms agreed to participate in the study and they were managed by experienced veterinarians and had high-level biosecurity measures.

Live bird markets are recognized as potential sources for the spread and transmission of pathogens [17]. The congregation of different species of poultry in these markets increases the chances for cross-contamination, a high diversity of pathogens and a possible emergence of new strains [18,19]. Poor hygiene and crowding facilitate the easy transmission and fast spread of pathogens via direct and indirect contact. Earlier studies have reported the presence of *S. aureus*, including MRSA, in live bird and chicken carcasses at poultry markets in Zaria [20] and Maiduguri [9] in northern Nigeria. A high prevalence (80.0%) of *S. aureus* was reported in samples from live chicken among small holder farms in Zaria [21], while Nworie et al. [8] reported a low MRSA prevalence (0.8%) in poultry farms in Ebonyi State, southeastern Nigeria.

There was diversity in the STs, the cgMLST clusters, *spa* and *dru* types among the 30 MRSA isolates subjected to WGS. All STs identified in this study are associated with the community-associated MRSA (CA-MRSA) involved in human infections. LA-MRSA clones were not detected in this study. Earlier studies have identified LA-MRSA ST398 in poultry meat [22,23], diseased poultry [24] or chickens at slaughter [25]. MRSA ST8 is a CA-MRSA with high diversity and has been reported in hospital and community settings in several African countries, including Nigeria [26,27]. MRSA ST8 with *spa* type t1476 identified in frozen chicken meat in this study had been reported in clinical samples from humans in Ghana [28]. ST121 has been described as highly disseminated CA-MRSA, and has been reported in Nigeria [7,29], as well as in other African countries [28,30]. All the ST121 isolates detected in the present study had *spa* type t314, similar to those reported in human healthcare institutions in Ghana [28]. In the present study, all the MRSA isolates from freshly dressed chicken in Ibadan belonged to ST152 with *spa* type t4690, which has also been reported in human clinical samples in Ghana [28], and from human cases of osteomyelitis in Ile-Ife, southwestern Nigeria [6]. MRSA ST772 has emerged as a CA-MRSA with a global spread [31,32]. All isolated MRSA belonging to ST772 in the present study were from human samples. ST772 has been reported in human infections in a tertiary care hospital in Southwest Nigeria [7]. The only isolate belonging to ST789 had *spa* type t091 and was recovered from frozen chicken in Abeokuta. A similar MRSA ST789 with *spa* type t091 was reported in hospital settings in Angola [33].

MRSA showed varying degrees of resistance to the tested antimicrobial agents. All MRSA isolates but one from humans were multidrug resistant. Multidrug resistance could be due to the co-selection of multiple resistance genes co-located on the same mobile genetic element following selective pressure induced by antimicrobial usage. An earlier study on the epidemiology of MRSA in Nigeria reported a high percentage of gentamicin, ciprofloxacin, and erythromycin resistance in MRSA of human origin [26], similar to the findings in the present study. However, in that study [26], MRSA isolates from humans also showed a high percentage of resistance to tetracycline, clindamycin and trimethoprim/sulfamethoxazole, contrary to the findings of the present study. In our study, the *mecA* gene was detected in all selected MRSA isolates. This gene was located in SCC*mec* cassettes of the types IVa, V or Vc. SCC*mec* elements of types IV and V are the most commonly encountered SCC*mec* types in CA-MRSA [27,34,35]. SCC*mec* elements of types IVa and V have been reported in hospital-associated MRSA (HA-MRSA) in Nigeria [29]. In addition to *mecA*, which confers resistance to a very broad range of β-lactams, a β-lactamase-encoding gene (*blaZ*) was detected among MRSA isolates from this study. Phenotypic resistance to tetracycline, gentamicin and erythromycin was supported by the detection of *tet*(K), *aphA3* and *msr*(A)/*mph*(C). All the tested MRSA harbored the gene *dfrG*, which confers resistance to trimethoprim. Moreover, most MRSA isolates in this study possessed *fosB* (resistance to fosfomycin) and one also possessed *sat4* (resistance to streptothricin), but our isolates were not investigated for phenotypic susceptibility to fosfomycin and streptothricin. The presence of these genes is likely to be explained by co-selection, as both antimicrobial agents are neither approved for nor used in chickens. The *sat4* gene has been described to be present in a resistance gene cluster consisting of the genes *aadE–sat4–aphA3* in *S. pseudintermedius* [36,37]. A further analysis showed that this cluster was not completely available in any of the sequenced MRSA isolates. Instead, a truncated cluster comprising *aadE–sat4–*Δ*aphA3* was detected in the single ST789/t091/dt11a isolate, whereas a different truncated cluster comprising *aadE–*Δ*sat4–*Δ*aphA3* was found in the seven ST772/t657/dt10ao isolates and in the two ST772/t657/dt10cj isolates.

The expression of virulence genes influences the pathogenicity of the respective isolates and the severity of staphylococcal infections [3]. All the MRSA isolates in this study possessed genes for virulence factors that play critical roles in adhesion, tissue destruction, invasion, immune modulation, erythrocytes and leucocyte lysis, and toxin production. The expression of these genes determines the pathogenesis and prognosis of MRSA infections [38]. Enterotoxins are responsible for staphylococcal food poisoning. All ST152/t4690 isolates had no enterotoxin genes, while MRSA of other STs possessed one or more of the enterotoxin genes *sea*, *seb* and *sec*. These enterotoxin genes are located on mobile genetic elements (MGEs), including bacteriophages (*sea*), plasmids (*seb*), or pathogenicity islands (*sec*) [39], which explains their presence or absence in individual isolates by the acquisition or loss of the respective MGEs. Many of the virulence genes detected in this study have been reported in CA-MRSA isolates from humans in hospital settings [40]. The diversity in virulence genes among the STs in the present study signifies variability in virulence-carrying plasmid and bacteriophages in the isolates [41,42]. The frequent occurrence of virulence genes in the MRSA isolates suggests that they may cause severe infections in susceptible hosts including humans. Thus, the presence of these potential pathogens in chicken meat, environmental sources and apparently healthy human carriers portends their role as a threat to public health.

## 4. Materials and Method

### 4.1. Sampling Locations and Sample Collection

**Figure 2:**
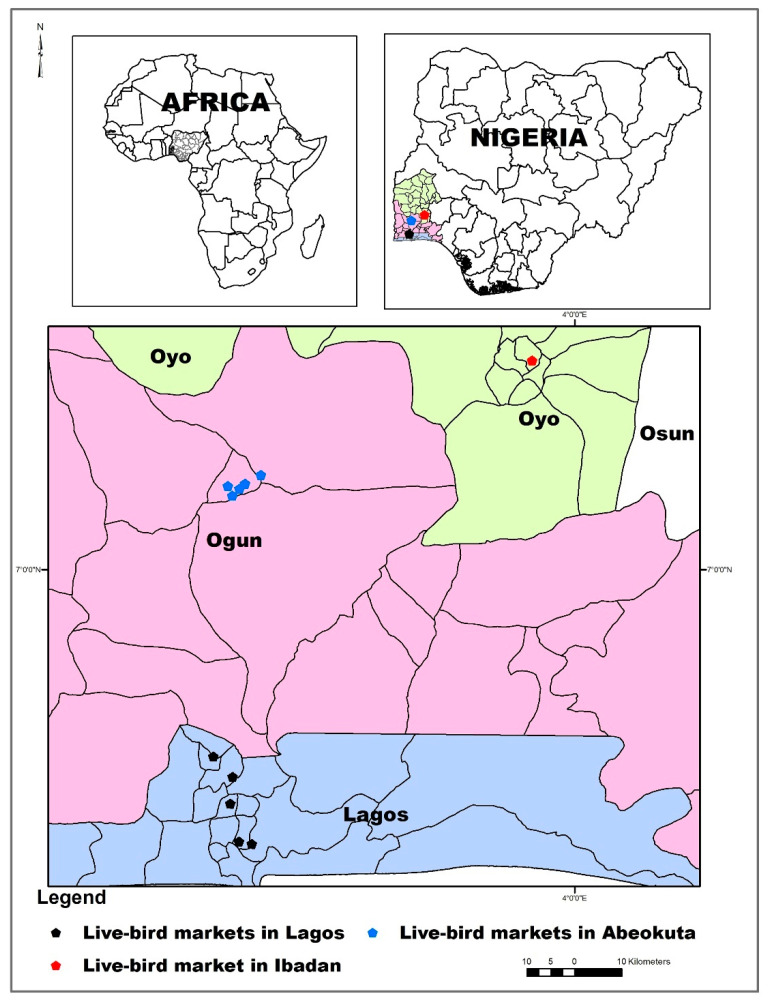
Sampling locations.

Samples were collected from human, chicken, chicken meat, and environmental sources within live bird markets and chicken slaughter centers in Lagos, Abeokuta and Ibadan, all located in the southwestern part of Nigeria (Figure 2).

Both Lagos and Ibadan have large markets for chicken products, including chicken meat. Ogun State with its capital Abeokuta is the largest producer of chickens in Nigeria. Farmers and middle men bring live chickens in large numbers from farms and supply these to vendors/retailers in the live bird markets. These vendors sell the chickens to individual consumers. It is very common for people to purchase chickens from vendors in live bird markets for household consumption. The chickens are slaughtered and processed (dressed) by processors within the market, either at the point of purchase or in nearby vicinity. Samples comprised nasal swabs from humans, meat of freshly dressed chicken carcasses, as well as a swab of the surface of utensils, such as knives and tables, used by chicken meat processors in slaughterhouses of purchase, or in the nearby vicinity. Samples comprised nasal swabs from humans and the meat of freshly dressed chicken carcasses, as well as swabs of the surface of utensils, such as knives and tables used by chicken meat processors in slaughterhouses (Table 6). Human samples were from chicken meat processors/vendors and consumers/buyers that purchased freshly dressed chicken meat from the slaughter centers. In addition, samples were collected from imported frozen chicken meat being sold within the premises of the live bird market. As a trace-back strategy, oropharyngeal swabs were collected from live chickens in three poultry farms that supplied live chickens to vendors at the live bird markets in Lagos and Abeokuta. Sampling was done within a five-month period from December 2016 to April 2017. Samples were labeled, preserved in icepacks and transported to the laboratory for microbiological analysis.

### 4.2. Isolation and Phenotypic Identification of Staphylococci Including S. aureus

Sample swabs were inoculated into 10 mL of Mueller–Hinton broth (MHB) containing 6.5% sodium chloride (NaCl) for pre-enrichment. Meat samples (25 g each) were thoroughly homogenized and inoculated into 225 mL of MHB for pre-enrichment. Pre-enrichment cultures were incubated at 35 ± 2 °C for 24 h. After the pre-enrichment stage, MHB cultures were streaked onto mannitol salt agar (MSA) with graded oxacillin supplementation; first at 2 mg/L and later at 4 mg/L. The MSA plates were incubated at 35 ± 2 °C for 24 to 48 h. The MSA plates were inspected for the presence of oxacillin-resistant, mannitol-fermenting colonies of bacteria. Oxacillin-resistant isolates were further sub-cultured on MSA supplemented with cefoxitin at 4 μg/mL. Cefoxitin-resistant isolates were noted. All oxacillin-resistant isolates (regardless of their cefoxitin resistance status) were selected and streaked onto blood agar (5% sheep blood) for the phenotypic identification of staphylococci. Identification tests included colony morphology and type of hemolysis on blood agar, Gram staining and microscopy, catalase, and oxidase, as well as coagulase and clumping factor tests. All oxacillin-resistant, catalase-positive, oxidase-negative Gram-positive cocci with cluster-like arrangement irrespective of coagulase production were selected for confirmatory tests to identify *S. aureus*. In total, 636 isolates, suspected to be staphylococci, were identified from 734 samples. Some samples yielded more than one suspected isolate, while others yielded none. Identified isolates were preserved on Tryptic Soy agar (supplemented with oxacillin) slants and refrigerated. For species identification and molecular characterization of isolates, preserved cultures were shipped to the laboratories at the Institute of Microbiology and Epizootics, Freie Universität (FU) Berlin, and the National Reference Laboratory for coagulase-positive staphylococci including *S. aureus* (NRL-Staph), Federal Institute for Risk Assessment (BfR), both located in Berlin, Germany.

Fresh staphylococcal colonies on blood agar were tested by MALDI-TOF mass spectrometry after an initial screening on Baird–Parker agar (BPA) for species identification. Isolates with inconclusive identification results after MALDI-TOF were further subjected to confirmation as *S. aureus* by an in-house multiplex real-time polymerase chain reaction (RT-PCR) assay, as described elsewhere [43] targeting the genus-specific gene *tuf* and the species-specific gene *nuc*. In parallel, and based on the presence/absence of the *mecA* gene coding for methicillin resistance, isolates were further identified as MRSA (*mecA*+) and MSSA (*mecA*−) genotypes, respectively.

### 4.3. Antimicrobial Susceptibility Testing

Of the 56 MRSA isolates, minimum inhibitory concentrations (MICs) were determined for 16 non-β-lactam antimicrobial agents or combinations of agents by broth microdilution, according to the recommendations of the CLSI [10]. For this, the microtitre plate layouts (Sensititre™), that were used in the national resistance monitoring program GE*RM*-Vet, were also used in this study. The antimicrobial agents tested and the test ranges in mg/L were as follows: ciprofloxacin (0.08–16), clindamycin (0.03–64), doxycycline (0.06–128), enrofloxacin (0.08–16), erythromycin (0.015–32), florfenicol (0.12–256), gentamicin (0.12–256), linezolid (0.03–64), nalidixic acid (0.06–128), neomycin (0.12–64), quinupristin/dalfopristin (0.015–8), streptomycin (0.25–512), tetracycline (0.12–256), tiamulin (0.03–64), trimethoprim/sulfamethoxazole (0.015/0.3–32/608), and vancomycin (0.015–32). *S. aureus* ATCC^®^29213 was included in the tests for quality control purposes.

### 4.4. DNA Extraction and Whole Genome Sequencing

Out of the 56 isolates confirmed as MRSA, 30 isolates were selected for WGS based on phenotypic resistance profiles, sample source and sampling locations. Representative isolates were selected among groups of isolates with similar phenotypic resistance profiles from different sample categories in each geographical location. Hence, all the identified phenotypic resistance profiles observed among isolates from different sample types within each geographical location were represented in the test collection submitted to WGS. All selected isolates had oxacillin MICs of ≥16 mg/L, suggesting that they were phenotypic MRSA.

*S. aureus* isolates grown in brain heart infusion (BHI) broth at 37 ± 2 °C for 24 h were harvested and DNA was extracted using the DNeasy Blood and Tissue Kit (Qiagen, Hilden, Germany). The quality of the DNA was evaluated by spectral analysis (NanoDrop Spectrophotometer, Thermo Fisher Scientific, Waltham, MA, USA), and the concentration was fluorometrically quantified by a Qubit 3.0 Fluorometer (dsDNA HS Assay Kit 0.2–100 ng; Thermo Fisher Scientific, Waltham, MA, USA). DNA libraries were prepared using the Nextera DNA Flex Library Prep Kit, according to the manufacturer’s instructions (Illumina, San Diego, CA, USA). Library quality was assessed using the fragment analyzer 3408 (Advanced Analytical Technologies Inc., Ames, IA, USA). Paired-end sequencing was performed on the Illumina NextSeq platform (2 × 151 cycles) using the NextSeq 500/550 Mid Output kit v2.5 (300 cycles).

Raw Illumina reads were trimmed and de novo assembled with our in-house developed AQUAMIS pipeline (https://gitlab.com/bfr_bioinformatics/AQUAMIS/), which implements fastp [44] for trimming, and shovill (based on SPAdes) (https://github.com/tseemann/shovill) for assembly. Furthermore, this pipeline performs Mash v 2.1 for reference search (http://genomebiology.biomedcentral.com/articles/10.1186/s13059-016-0997-x), as well as QUAST v 5.0.2 for assembly quality control (https://github.com/ablab/quast). The Whole Genome Shotgun project has been deposited at DDBJ/ENA/GenBank under the accession numbers JABUZL000000000-JABVAO000000000. To determine the phylogenetic relationships, sequences were analyzed by Ridom Seqsphere+ v. 6.0.0 (April 2019) (Ridom, Muenster, Germany), using the proposed cgMLST scheme of 1783 gene targets [45], with 98% required identity and 98% required percentage of coverage to one of the known alleles (allele library status December 2019). Bacterial characterization was conducted with our in-house developed BakCharak pipeline (https://gitlab.com/bfr_bioinformatics/bakcharak), which implements ABRicate (https://github.com/tsmeeann/abricate) for the screening of antimicrobial resistance genes (using the NCBI AMRFinder database [46]), plasmid markers (using the PlasmidFinder database [47]) and virulence factors (using the VFDB database (https://dx.doi.org/10.1093%2Fnar%2Fgki008)). In addition, the ResFinder 4.0 database (https://cge.cbs.dtu.dk/services/ResFinder/) was used for identification of antimicrobial resistance genes and chromosomal mutations conferring antimicrobial resistance. The *spa* (https://spaserver.ridom.de/), *dru* (http://dru-typing.org/), and MLST (https://pubmlst.org/bigsdb?db=pubmlst_saureus_seqdef) types were deduced from the whole genome sequences.

## 5. Conclusions

Live bird markets are likely focal points for the dissemination of CA-MRSA of different STs. Chicken meat in these markets was contaminated with MRSA of different STs from unidentified sources, and may serve as vehicle for the transmission of MRSA in the community. Although there was no evidence of the direct exchange of MRSA among the various sample sources, the high diversity of MRSA isolates within the markets may facilitate the distribution of different MRSA isolates within the market environment. Over time, these MRSA isolates may cross the barrier of host specificity, leading to exchanges among different sources and the adaptation of specific MRSA types to new hosts.

## Figures and Tables

**Table 1:** Distribution of MRSA isolates from different sample types and sampling locations.

Sample Categories	Sample Locations and Sample Size			
	Abeokuta	Ibadan	Lagos	Total
Freshly dressed chicken	13	15	0	28
Frozen/imported chicken	2	0	0	2
Processors	10	0	0	10
Consumers	0	0	8	8
Knives	2	0	0	2
Tables	0	6	0	6
Total	27	21	8	56

**Table 2:** Minimum Inhibitory Concentrations of non-β-lactam antimicrobial agents of the 56 MRSA isolated from live bird markets in Nigeria.

Antimicrobial Agent (s)	Number of Isolates with MIC Value (mg/L) Is																
	0.008	0.015	0.03	0.06	0.12	0.25	0.5	1	2	4	8	16	32	64	128	256	512
Vancomycin (VAN)		-	-	-	-	-	-	51	5	-	-	-	-				
Florfenicol (FFC)					-	-	-	-	-	47	9	-	-	-	-	-	
Erythromycin (ERY)		-	-	-	-	16	24	-	-	-	3	12	1				
Clindamycin (CLI)			-	3	51	2	-	-	-	-	-	-	-	-			
Streptomycin (STR)						-	-	-	-	4	39	12	1	-	-	-	-
Gentamicin (GEN)					1	13	16	6	2	-	-	-	2	6	5	3	2
Neomycin (NEO)					1	15	22	1	-	-	-	-	-	-	17		
Ciprofloxacin (CIP)	-	-	-	-	-	2	28	6	1	-	3	14	2				
Enrofloxacin ^1^ (ENR)	-	-	-	-	12	18	7	-	-	13	5	-	1				
Nalidixic acid (NAL)				-	-	-	-	-	-	-	-	1	5	27	4	19	
Trimethoprim/sulfamethoxazole (1:19) *^c^* (SXT)		-	-	-	-	1	7	30	12	4	-	1	-	1			
Tetracycline (TET)					-	6	12	4	-	-	-	-	6	26	1	1	
Doxycycline (DOX)				-	13	9	1	-	5	23	5	-	-	-	-		
Linezolid (LZD)			-	-	-	-	-	14	37	5	-	-	-	-			
Tiamulin (TIA)			-	-	-	2	32	22	-	-	-	-	-	-			
Quinupristin/dalfopristin (Q/D)		-	-	-	-	29	26	1	-	-	-						

The grey shaded areas are the test ranges not included in the test panels for the respective antimicrobial agents. Isolates that had no growth in any of the concentrations are given the lowest MIC value. Isolates with growth in all the tested concentrations are given next serially higher MIC value above the highest tested concentration. *^c^* The MIC values of trimethoprim/sulfamethoxazole (1:19) are expressed as the MIC values of trimethoprim. The colors indicate the following classifications: susceptible—green; intermediate—yellow; resistant—red. ^1^ The breakpoints for enrofloxacin are applicable to *Staphylococcus* spp. from cats and were taken from the CLSI document VET08 4th ed. [12] while all other clinical breakpoints are applicable to humans and were taken from CLSI document M100 30th ed. [13]; there are currently no CLSI-approved clinical breakpoints for florfenicol, streptomycin, neomycin, nalidixic acid, and tiamulin applicable to staphylococci.

**Table 3:** Antimicrobial resistance phenotypes and molecular characteristics of the whole genome-sequenced MRSA isolates.

ID	Source	Resistance Profile *	*spa*	*dru*	SCC*mec*	MLST
118	FDC/Abeokuta	BLA	t314	Nt	IVa	ST121
119	FDC/Abeokuta	BLA-SXT-TET	t314	Nt	IVa	ST121
128	FDC/Abeokuta	BLA-TET	t314	Nt	IVa	ST121
130	FDC/Abeokuta	BLA	t314	Nt	IVa	ST121
134	FDC/Abeokuta	BLA-TET	t314	Nt	IVa	ST121
138	FDC/Abeokuta	BLA	t314	Nt	IVa	ST121
139	FDC/Abeokuta	BLA-TET	t314	Nt	IVa	ST121
F16	FDC/Abeokuta	BLA-TET	t314	Nt	IVa	ST121
141	FDC/Ibadan	BLA-SXT-TET	t4690	dt11a	Vc	ST152
142	FDC/Ibadan	BLA-SXT-TET	t4690	dt11a	Vc	ST152
145	FDC/Ibadan	BLA-ERY-TET	t4690	dt11a	Vc	ST152
146	FDC/Ibadan	BLA-SXT-TET	t4690	dt11a	Vc	ST152
153	FDC/Ibadan	BLA-TET	t4690	dt11a	Vc	ST152
156	FDC/Ibadan	BLA-TET	t4690	dt9aw	Vc	ST152
157	FDC/Ibadan	BLA-TET	t4690	dt11a	Vc	ST152
190	FIC/Abeokuta	BLA-CIP-GEN-TET	t1476	dt10dr **	Unknown	ST8
202	FIC/Abeokuta	BLA-CIP-GEN-TET	t091	dt11a	V	ST789
244	HNP/Abeokuta	BLA-CIP-SXT-TET	t2331	dt11dw **	Vc	ST8
250	HNP/Abeokuta	BLA-CIP-ERY-GEN	t657	dt10ao	V	ST772
253	HNP/Abeokuta	BLA-CIP-ERY-GEN	t657	dt10ao	V	ST772
254	HNP/Abeokuta	BLA-CIP-ERY-GEN	t657	dt10ao	V	ST772
257	HNP/Abeokuta	BLA-CIP-ERY-GEN	t657	dt10ao	V	ST772
376	HNC/Lagos	BLA-CIP-ERY-GEN	t657	dt10cj	V	ST772
381	HNC/Lagos	BLA-CIP-ERY-GEN	t657	dt10ao	V	ST772
382	HNC/Lagos	BLA-CIP-ERY-GEN	t657	dt10cj	V	ST772
385	HNC/Lagos	BLA-CIP-ERY-GEN	t657	dt10ao	V	ST772
399	HNC/Lagos	BLA-CIP-ERY-GEN	t657	dt10ao	V	ST772
432	ETS/Ibadan	BLA-TET	t12236	dt11a	Vc	ST8
434	ETS/Ibadan	BLA-TET	t12236	dt11a	Vc	ST8
481	EKS/Abeokuta	BLA-TET	t12236	dt9aw	Vc	ST8

LEGEND: FDC: Freshly dressed chicken; FIC: Frozen/imported chicken; HNP: Human nasal swab, processor; HNC: Human nasal swab, consumer; ETS: Environment, table swab; EKS: Environment, knife swab; ST: Sequence type; BLA: β-lactam resistance; * For abbreviations of antimicrobial agents, please refer to Table 2; Nt: not typeable; ** dt10dr and dt11dw are novel *dru* types, first described in this study.

**Table 4:** Virulence genes detected in specific whole genome-sequenced MRSA isolates.

Genes Encoding Virulence Factors	Present
*cap8 H*, *I*, *J*, *K* (capsular polysaccharide)	All ST121 and ST789 isolates
*chp* (chemotaxis inhibitory protein)	Only in one isolate (ST8, t1476)
*clfA* (clumping factor A)	All isolates but one (ST152/t4690)
*clfB* (clumping factor B)	All but four isolates (ST152/t4690, ST8/t2331, ST8/t12236 and ST772/t657)
*cna* (collagen adhesin)	All ST152, ST772 isolates and one ST121 isolate
*ebp* (elastin binding protein)	All ST8, ST152, ST789 and ST772 isolates
*esaC* (Type VII secretion system)	All ST8, ST121, ST152, ST789 isolates
*esxB* (Type VII secretion system)	All ST8, ST121, ST152, ST789 isolates
*fnbB* (fibronectin binding protein)	All but three isolates (ST772)
*hysA* (hyaluronidase)	All ST8, ST121, ST152, ST772 isolates
*lukF-PV* (Panton-Valentine leukocidin)	All ST121, ST152 except one isolate (157), ST772 isolates
*lukS-PV* (Panton-Valentine leukocidin)	All ST121, ST152 except one isolate (157), ST772 isolates
*lukD* (Leukotoxin D)	All ST8, ST121, ST152, ST789 isolates
*lukE* (Leukotoxin E)	All ST8, ST121, ST152, ST789 isolates
*map* (MHC class II analog protein)	All ST8, ST121, ST772, ST789 isolates
*sak* (staphylokinase)	All ST8, ST121, ST152, ST789 isolates
*sdrC* (serine aspartate repeat protein)	All ST8, ST121, ST772, ST789 isolates
*sdrD* (serine aspartate repeat protein)	All ST8, ST121, ST772, ST789 isolates
*sdrE* (serine aspartate repeat protein)	All but one isolate (ST8/t2331)
*sea* (staphylococcal enterotoxin)	All ST772, ST789 and four ST8 isolates
*seb* (staphylococcal enterotoxin)	All ST121 isolates
*sec* (staphylococcal enterotoxin)	Only in five isolates (ST772)
*sell* (staphylococcal enterotoxin-like)	Only in five isolates (ST772)

**Table 5:** Resistance gene profiles of the 30 whole genome-sequenced MRSA isolates.

Profile	Resistance Genes Present	MLST/*spa*/*dru* Type (Number of Isolates)
1	*mecA-dfrG-tet*(K)*-tet*(38)	ST152/t4690/dt11a (4) or ST152/t4690/dt9aw (1)
2	*mecA-blaZ-dfrG-fosB-tet*(38)	ST121/t314/nt (3)
3	*mecA-blaZ-dfrG-tet*(K)-*tet*(38)	ST152/t4690/dt11a (2)
4	*mecA-blaZ-dfrG-fosB-tet*(K)*-tet*(38)	ST8/t12236/dt11a (2), ST8/t12236/dt9aw (1), and ST121/t314/nt (5)
5	*mecA-blaZ-dfrG-fosB-tet*(38)-*msr*(A)*-mph*(C)-*aacA/aphD-aphA3*-Δ*sat4*-Δ*aadE*	ST772/t657/dt10ao (7), ST772/t657/dt10cj (2)
6	*mecA-blaZ-dfrG-fosB-tet*(K)-*tet*(38)	ST8/t2331/dt11dw (1)
7	*mecA-blaZ-dfrG-dfrS1-fosB-tet*(K)-*tet*(38)-*aacA/aphD*	ST8/t1476/dt10dr (1)
8	*mecA-blaZ-dfrG-tet*(K)-*tet*(38)-*aacA/aphD-aphA3-sat4*-Δ*aadE*	ST789/t091/dt11a (1)

**Table 6:** Sample size by categories and sampling locations.

Sample Categories	Sample Locations and Sample Size			
	Abeokuta	Ibadan	Lagos	Total
A. On-farm				
Live chicken	68	-	74	142
B. Live bird markets (meat samples)				
Freshly dressed chicken	20	5	33	58
Frozen/imported chicken	27	-	63	90
C. Live bird markets (human samples)				
Processors	12	5	15	32
Consumers	34	-	245	279
D. Live bird markets (environmental samples)				
Knives	6	-	53	59
Tables	16	5	53	74
Total	183	15	536	734
